# Mean Nasal Index of Dental Students of a Dental College in Nepal

**DOI:** 10.31729/jnma.4208

**Published:** 2019-04-30

**Authors:** Ritee Shrestha, Bipana Manandhar, Hari Prasad Upadhyay, Nirjala Laxmi Madhikarmi

**Affiliations:** 1 Department of Anatomy, Kantipur Dental College, Basundhara, Kathmandu, Nepal; 2 Department of Community Medicine, College of Medical Sciences, Bharatpur, Chitwan, Nepal; 3 Department of Biochemistry, Kantipur Dental College, Basundhara, Kathmandu, Nepal

**Keywords:** *mesorrhine*, *nasal height*, *nasal index*, *nasal width*, *nose type*

## Abstract

**Introduction:**

Human nose is one of the important anthropometric parameters for identification of sex and ethnicity of an individual of an unknown identity. The nasal index holds a great value in anthropological studies, because it is one of the anthropometric indices acknowledged in nasal surgery as well as management. The study aims to find the mean nasal index and the nose type of dental students of Kantipur Dental College of Nepal.

**Methods:**

The descriptive cross-sectional study was conducted among 140 dental students in the Department of Anatomy, Kantipur Dental College Teaching Hospital and Research Center, Basundhara, Kathmandu. The study population belongs to dental students of Kantipur Dental College and Research Center. The nasal parameters include nasal height, nasal width which was measured using Digital Vernier Caliper and the nose was classified in three different types based on the value of nasal index.

**Results:**

The mean nasal index of total population was 81.34±14.88 mmwith confidence interval range of 78.85 and 83.83 mm. Mesorrhine type of nose was found to be most common among the total population. Mean nasal index in male is 84.49±12.46 mm and in female is 80.66±15.32 mm.

**Conclusions:**

This study concludes that the mean values of nasal index of the students fall under mesorrhine type of nose.

## INTRODUCTION

Nasal anthropometry is the study concerned with the measurements of different parameters of the human nose. It is one of the features of the body that has been reported to be ethnicity specific and gender specific.^1^

Nasal index (NI) is considered to be one of the finest clues of nasal parameter to distinguish different races, ethnicity and gender of an individual and is a vital measurement in performing the rhinoplasty and even in the evaluation and diagnosis of craniofacial deformities. ^2^ It is one of the methods anthropologists have used to differentiate living race and subspecies of man.^3^ On its basis, the nose shape can be classified into leptorrhine or fine nose, mesorrhine or medium nose, platyrrhine or broad nose.^4^

The main objective of this study is to find the mean nasal index and nose type of dental students of Kantipur Dental College and Research Center.

## METHODS

A descriptive cross-sectional study was conducted in the Department of Anatomy, Kantipur Dental College and Research Centre, Kathmandu, Nepal. After obtaining ethical approval from IRC, data was collected from 140 students of Kantipur Dental College from September to December 2018. The main reason for the selection of adults as study group was due to the fact that the morphology of human body is not stable during earlier years of life and gets stable overtime only during adulthood. Verbal consent was obtained from each students and the purpose of the research was conveyed to them. The individuals were of age group 17–25 years. The subject comprised of individuals with normal craniofacial configuration and without any past surgery of head and neck. Subjects with trauma of the craniofacial structures and congenital abnormalities were excluded.

Sample size was calculated using a formula;

Where,

n = required sample size
Z = 1.96 at 95 % confidence interval
SD= standard deviation (0.3)
e = margin of error (5%)

Standard deviation mentioned was 0.3 according to educated guess following which sample size derived was 138.29. Altogether, 140 students were enrolled in the study by convenient sampling method. The instrument used in the study was a Digital Vernier Caliper with accuracy of 0.01 mm. Subjects were told to sit upright in a relaxed mood with head in an anatomical position and without any facial expression, so that it does not alter the size of nose. The measurements taken were in millimetres. Following three relevant nasal surface landmarks were identified on the subjects with careful inspection and then marked on nose with black marker.

1. Nasion: the point at which the nasal suture meets the fronto-nasal suture.
2. Subnasal: the point where nasal septum and the upper lip meet in the mid-sagittal plane.
3. Alae: the expanded outer wall of nasal cartilage on each side of nose.

The measurements were taken that includes:

1. Nasal height (NH): distance from nasion to subnasale.
2. Nasal width (NW): distance from ala to ala, at right angle to nasal height.
  And the value of NI was determined based on the formula,
$$\begin{array}{lll} \text{n} & = & {\text{Z}}^{2}\times {\text{SD}}^{2}/{\text{e}}^{2}\\ & = & {\left(1.96\right)}^{2}\times {\left(0.3\right)}^{2}/{\left(0.05\right)}^{2}\\ & = & 138.29 \end{array}$$

Then, the nose type was classified on the basis of nasal index as:

1. Leptorrhine: NI<69.9
2. Mesorrhine: NI=70–84.9
3. Platyrrhine: NI>85

The data was entered and analyzed with the help of SPSS 20 software. The descriptive data analysis was done to find mean and standard deviation of nasal width, nasal height and nasal index within the study participants.

## RESULTS

The present study comprised of 140 dental students. The mean nasal index of total population was found to be 81.34± 14.88 mm with confidence interval range of 78.85 and 83.83 mm. The mean nasal index of total population was 81.34± 14.88 mm with confidence interval range of 78.85 to 83.83 mm. Mesorrhine type of nose was found to be most common among the total population.

Subgroup analysis was done among two genders comprising of 25 male and 115 female students. The mean value of all three nasal parameters including NW, NH and NI were calculated using Digital Vernier Caliper in all individuals (Table 1). The unit for the measurement of all three parameters were in millimetre (mm), as mentioned in the instrument. The mean nasal index of total population was 81.34± 14.88 mm with confidence interval range of 78.85 to 83.83 mm. Mesorrhine type of nose was found to be most common.

**Table 1:** Overall comparison of all three nasal parameters (n= 140).

					95 % CI for	
Nasal param eters	Mean (mm)	SD (mm)	Maxi mum (mm)	Maxi mum (mm)	Mean Lower (mm)	Upper (mm)
NW	44	4.35	34.24	61.34	43.27	44.72
NH	54.81	5.08	38.59	65.82	53.96	55.66
NI	81.34	14.88	63.09	142.4	78.85	83.83

The mean of all three parameters were determined in two genders (Table 2). All the parameters were found to be greater in male as compared to female, which suggest that there occurs a sexual dimorphism in all three parameters.

**Table 2:** Mean and SD of nasal parameters in two different genders (n_1_ = 25, n_2_=115).

Nasal parameters (Mean±SD)	Male (n_1_)	Female (n_2_)
Nasal Height (mm)	55.56±4.29	54.66±5.25
Nasal Width (mm)	46.56±4.05	43.44±4.22
Nasal index (mm)	84.49±12.46	80.66±15.32

## DISCUSSION

According to our study, the mean NH, NW and NI were found to be 44±4.35 mm,54±5.08 mm and 81.34 ±14.88 mm respectively. Mesorrhine type of nose was found to be most common. Similarly, the mean NH, NW and NI were found to be 55.56± 4.29 mm, 46.56±4.05 mm and 84.49± 12.46 mm respectively in male and 54.66±5.25 mm, 43.44± 4.22 mm and 80.66±15.32 mm in female respectively. It is concluded based on the results of the present study that noses in dental students of male and female shows sexual dimorphism with specific masculine and feminine anthropometric features. Based on the value of NI, mesorrhine type of nose was found to be most common among both the genders. In a study done by Rusetskii YY et al. in Kazakh population, the mean NI were found to be 80.8±0.53 in male and 79.4±3.17 in female, which value were less than the values obtained in our body for both male and female. But the type of nose according to the value of NI was mesorrhine type of nose which was similar to our findings.^5^ According to Ikechukwu CE et al the NI in Ibo male and female were 107.62 ±1.09 and 98.89+1.30 respectively and in Yoruba male and female were 110.3to1.92 and 97.07 ±1.11 respectively. In contrast to our study, the value of NI in both the races of Nigeria shows much higher than the NI of our population and the type of nose was platyrrhine.^6^ In a study conducted by Oladipo G, in two different ethinicity of Nigeria, mean nasal width for Urhobo male and female was found to be 39.15±3.5 mm and 36.83 ± 3.56 mm respectively and Itsekiri male and female was found to be 38.60±3.64 mm and 36.28± 2.6 mm. The mean nasal height for Urhobo male and female was found to be 46.67±1.23mm and 41.51±3.52 mm respectively and Itsekiri male and female was found to be 42.02±3.52 and 40.83±3.29 mm,^7^ whose value was less of both the nasal width and nasal height compared to our study in both genders. Similarly in the study carried out by Zolbin MM, the mean nasal width of students of Qazvin University of Medical Sciences, Iran was found to be 38.0± 2.3 mm and 33.80± 2.20 mm respectively in male and female, and the nasal height was 60.6± 2.9 mm and 56.80± 2.80 mm in male and female respectively. It suggests that the mean value of nasal width is less but that of nasal height in more in Irish students as compared to Nepalese students.^8^

In contrast to the study conducted by Oladipo G, nasal index in three different indigenous people of Africa including Igbos, Yorubas and Ijaws, was found to be greater than 85.0 and found to be highest in Igbos being 96.37 ±1.06 and least in yorubas being 89.2 ±0.3. All three ethnic groups have platyrrhine nose as accepted of an African population, which was further proved by Bantus and Bushmen, both the people of Africa and indigenous Australian has nasal index above or equal to 85.0 with platyrrhine type of nose.^9^ Most of the Europeans have Nasal index 69.9 or less with leptorrhine type of nose, which was similar to nose type of german.^10^ It can also be stated that leptorrhine type of nose is predominant in most of European. Similary, in a study carried out by Staka G, in Dental students of University of Prishtine, Albania, the prevalent nose type was found to be leptorrhine being 67.33% and 86.40% in male and female respectively. ^11^

In the studies demonstrated by Ikechukwu CK in Iblo^6^ and Oladipo G in Yoruba,^12^ both ethnicity have basically platyrrhine type of nose which is in conformity with the work of Oladipo Gsouthern Nigeria. ^13^

The value of nasal index in total population in our study were 84.49±12.46 and 80.66±15.32 respectively in male and female which was in accordance with the study carried out in Nepal by Koirala S. Nasal index in mongloid male and female was 74.6 and 75.9 respectively and in tharu male and female was 83.8 and 82.4 respectively. It suggested that both mongloid and tharu have mesorrhine type of nose which was in accordance with our findings in both genders.^14^ Mohammed I suggested that the nasal index in bekwara male and female was 94.65±6.4 mm and 90.33±6.4 mm respectively i.e male has higher nasal index than female.^15^ This further illustrates the racial as well as sexual differences in nasal parameters. Similarly in all ethnic groups male has higher nasal index than female with statistically significant differences which was similar to our findings.

The parameters of the external nose in present study may be of value not only in terms of reconstructive and aesthetic rhinosurgery but also for the purpose of forensic medical and other expertises. Nasal index is very useful in anthropology in distinguishing gender and ethnic differences.

The limitation of our study includes the less number of subjects with unequal distribution of male and female. The sampling was done by convenient sampling technique so the result cannot be generalised. Although all the measurements were done meticulously there might be slight difference measurements obtained by other person. To increase the validity of result, random sampling must be done with larger population setting.

## CONCLUSIONS

This study concludes that the mean values of nasal index of all the students fall under mesorrhine (medium) type of nose and there was no much difference in the nasal index in male and female.
